# Turning over New Leaves

**Published:** 1889-07-27

**Authors:** 


### Turning Over New Leaves.

### SONGS OF THE SPINDLE.*

An artistic little book lies on our table, which, as the prefatory note explains, is the product of "hand-work alone." In these days of haste and cheap production, such a book is rare, and possesses, in consequence, a peculiar interest. The paper was made by hand, the printing done by a hand-press, the flax—which forms the basis of both linen cover and the paper leaves—was spun by the cottagers at their wheels in the Langdale Valley, and the thread used for the cover was woven on a hand-loom in the same place. The selector and editor considers, and not without ground, that books produced in this way have an individuality not possessed by those which are turned out by machinery in thousands. The poems, which wander down a luxurious margin of thick tinted paper, comprise spinning and weaving songs stretching over a period of three thousand years, and there are eleven illustrations. We understand that the edition is limited, and that each copy will be numbered.

* "Songs of the Spindle and Legends of the Loom." Selected and arranged by H. H. Warner. London: N. J. Powell and Co.

